# Sympathetic neurons’ contribution to arrhythmias in a hiPSC‐based myotonic dystrophy type 1 microphysiological system

**DOI:** 10.1002/ctm2.70860

**Published:** 2026-10-27

**Authors:** Jean‐Baptiste Reisqs, Yvonne Sleiman, Mohamed Boutjdir

**Affiliations:** ^1^ Cardiovascular Research Program VA New York Harbor Healthcare System New York New York USA; ^2^ Department of Medicine, Cell Biology and Pharmacology State University of New York Downstate Health Sciences University New York New York USA; ^3^ Division of Cardiology, Department of Medicine NYU Grossman School of Medicine New York New York USA

To the Editor:

Myotonic dystrophy type 1 (DM1) is a multi‐systemic neuromuscular genetic disorder, characterized by progressive muscle wasting, myotonia, dystrophic changes in skeletal and cardiac muscles, and the nervous system.^1^ The underlying cause of DM1 is an expansion of cytosine, thymine, and guanine (CTG) repeats within the dystrophia myotonica‐protein kinase (DMPK) gene.^2^ Cardiac complications are a critical factor in DM1, leading to sudden cardiac death, and ranking as the second most common cause of death after respiratory complications,^1^ with up to 80% of patients experiencing cardiac electrical abnormalities.^1^ Primary cardiac manifestations involve disruptions in the heart's conduction system, frequently leading to conduction block and arrhythmia and later to heart failure.

The autonomic nervous system (ANS) plays a significant role in the initiation and maintenance of arrhythmias in DM1, as an imbalance in autonomic tone generally constitutes an arrhythmogenic substrate.^3^ The ANS branches include the sympathetic nervous system (SNS) and parasympathetic nervous system. Clinical evidence has shown that patients with DM1 exhibit ANS dysfunction, as evidenced by an inverse correlation between heart rate variability (HRV) parameters and PR interval, demonstrating a correlation between ANS disturbance and ECG abnormalities.^4^ We hypothesized that ANS dysfunction contributes to arrhythmias in DM1 patients.

Here, we investigated, for the first time, the electrophysiological properties of sympathetic neurons and their impact on cardiomyocytes from the same DM1 patient. We developed a co‐culture method to study the neuro‐cardiac junction (NCJ) based on the use of a compartmentalized microfluidic device (MFD).^5^, ^6^ Human induced pluripotent stem cells (hiPSCs) from a DM1 patient with 700 CTG repeats and a control (sex‐ and age‐matched) were used (CancerTools and SCVI Biobank). The protocol used for the sympathetic neuronal differentiation was previously validated.^7^ We performed patch‐clamp experiments to study spontaneous electrical activity of sympathetic neurons (hiPSC‐SNs) and cardiomyocytes from hiPSCs (hiPSC‐CMs) in monoculture and co‐culture in the MFD. For the co‐culture, the hiPSC‐SNs were seeded into the neuronal compartment for axonal growth into the adjacent chamber. hiPSC‐CMs were added to the second compartment, and experiments were performed 10 days post co‐culture.

We validated the sympathetic phenotype of both control and DM1 hiPSC‐SNs (Figure 1A,B). These neurons were positive for peripherin and tubulin‐3, both structural proteins, and tyrosine hydroxylase, an enzyme implicated in norepinephrine synthesis, in both control and DM1 hiPSC‐SNs, validating the efficiency of neuronal differentiation (Figure 1B). Furthermore, equivalent morphological maturation was found between control and DM1 neurons (Figure S1). We then examined action potential (AP) firing patterns in response to prolonged (250 ms) steps with increasing current injection amplitude in both control and DM1 hiPSC‐SNs (Figure 1C). Neuronal firing behaviour was classified into three patterns: Phasic 1 representing neurons with only one AP; Phasic 2 neurons firing 2–4 APs; and Phasic 3 tonic‐firing neurons > 5 APs (Figure 1D). Interestingly, DM1 hiPSC‐SNs were found to be predominantly tonic‐firing (60% of cases), while controls exhibited only 30% of tonic firing (Figure 1E). We also measured APs of hiPSC‐SNs at 1 Hz to measure afterhyperpolarization (AHP) properties in control and DM1 (Figure 1F). AHP parameters were decreased in DM1 hiPSC‐SNs, including AHP duration (259 ± 36 ms vs. 123 ± 15 ms) (Figure 1G), AHP amplitude (−6 vs. −4 mV) (Figure 1H), and the global area of AHP (870 ± 111 mV.ms vs. 576 ± 95 mV.ms) (Figure 1I) demonstrating a hyperexcitability of DM1 hiPSC‐SNs.

**FIGURE 1:**
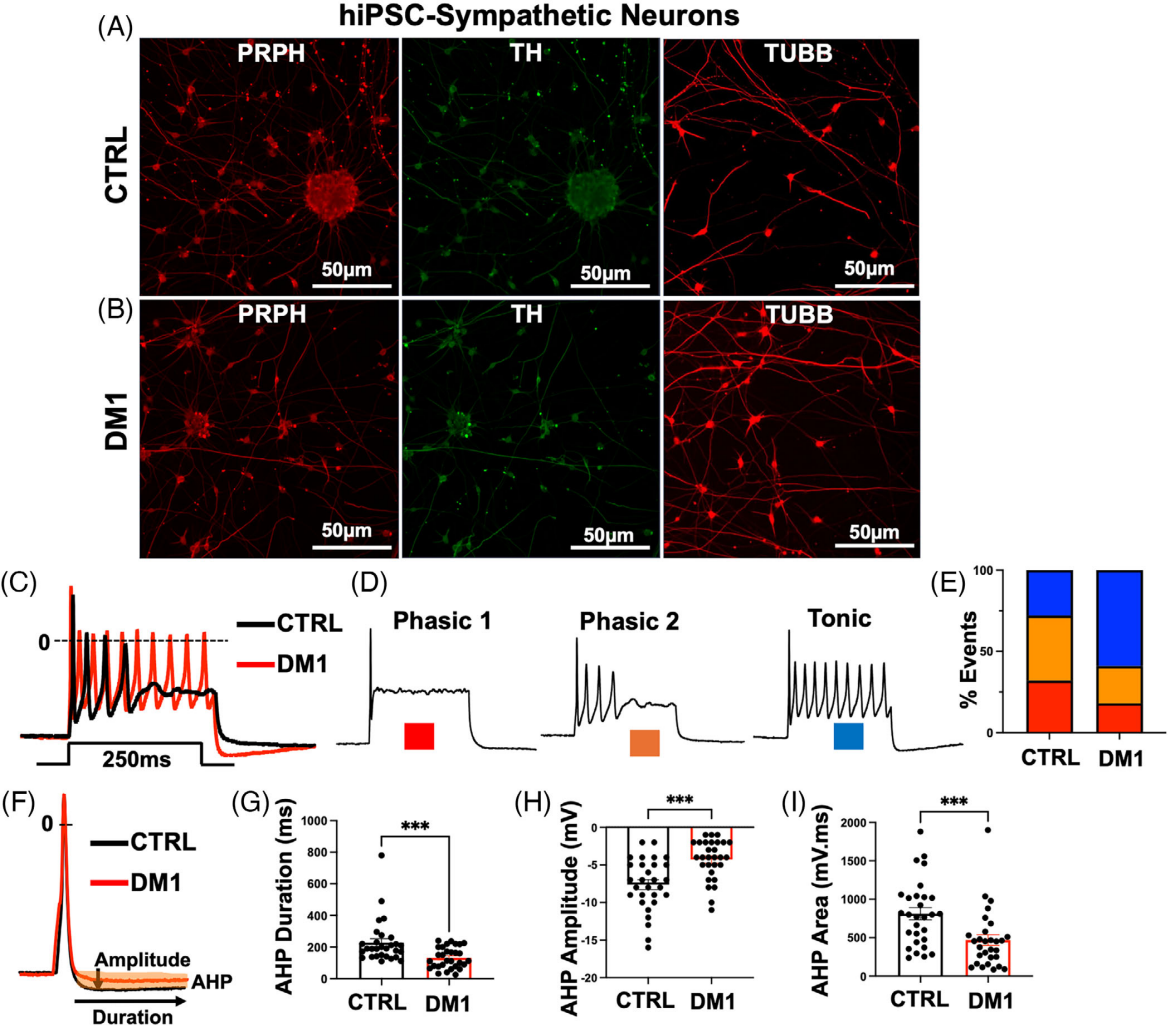
Morphology and functional phenotype of DM1 versus hiPSC‐sympathetic neurons. Immunostaining experiments were performed in hiPSC‐SNs derived from a control (CTRL) and a DM1 (700 CTG repeats). (A, B) CTRL and DM1 hiPSC‐SNs positive for peripherin (PRPH), tyrosine hydroxylase (TH), and beta‐III tubulin (TUBB), validating the cells as catecholaminergic neurons in the peripheral nervous system. (C) Representative traces of action potential (AP) firing frequencies in response to prolonged current in CTRL (black) and DM1 (red) hiPSC‐SNs. (D) Examples of neuronal firing behaviour subtypes, including phasic 1 (red), phasic 2 (orange), and tonic firing (blue), indicated by a coloured square, and (E) percentage calculation of firing pattern comparison between CTRL and DM1 hiPSC‐SNs. (F) Representative individual AP traces used to measure afterhyperpolarization (AHP) properties, with (G) duration, (H) amplitude, and (I) the global area of AHP. Mann‐Whitney test was performed; means ± SEM; *p < .05, **p < .01, ***p < 0.001. CTRL *n* = 29; DM1 *n* = 30 from three independent differentiations.

We then explored the electrophysiological properties of DM1 hiPSC‐CMs by measuring AP duration (APD) paced at 1 Hz. DM1 hiPSC‐CMs showed a prolongation of APD at 90% (558 ± 24 ms vs. 435 ± 19 ms) and 50% (462 ± 25 ms vs. 300 ± 18 ms) of repolarization (Figure 2A–C). In addition, DM1 hiPSC‐CMs exhibited a higher APD alternans compared with control (Figure 2D).

**FIGURE 2:**
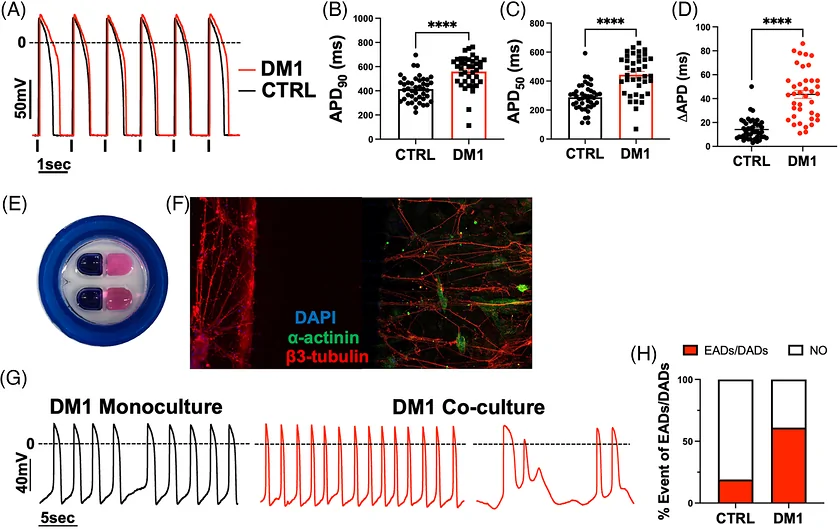
Functional phenotype of DM1 hiPSC‐derived cardiomyocytes in monoculture and co‐culture. (A) Examples of APs hiPSC‐CMs in control (CTRL) (black; *n* = 43) and DM1 (red; *n* = 39) hiPSC‐CMs recording during patch‐clamp experiments paced at 1 Hz. The measures of action potential duration at (B) 90% (APD_90_) and (C) 50% (APD_50_) of repolarization show a significant increase in APD in DM1 hiPSC‐CMs. (D) Calculation of ΔAPD (APD_long_–APD_short_) showing an increase in APD alternans in DM1 hiPSC‐CMs. (A–D) Results are from three independent differentiations. (E) Representation of the microfluidic device, composed of two independent neuro‐cardiac co‐cultures, with one neuronal chamber (blue) connected to the cardiomyocyte chamber (pink) by microchannels. (F) Immunohistochemical staining of hiPSC‐SNs (red = β3‐tubulin) connected to hiPSC‐CMs (green = α‐actinin); blue corresponding to DAPI; Magnification ×20. (G) Examples of spontaneous APs hiPSC‐CMs from DM1, recorded during gap‐free mode, in monoculture (black; *n* = 12) and co‐cultured for 10 days with hiPSC‐SNs in a microfluidic device (red; *n* = 11), showing an increase in spontaneous APs and electrical aberrant activity (early afterdepolarizations (EADs) or delayed afterdepolarizations (DADs)). (H) Calculation of the percentage incidence of EADs and DADs in CTRL (*n* = 9) versus DM1 (*n* = 11) hiPSC‐CMs. Mann–Whitney test was performed; mean ± SEM; ****p < .0001. Results are from three independent differentiations.

To examine how DM1 hiPSC‐SNs could exacerbate the electrical activity of DM1 hiPSC‐CMs, we established a co‐culture system using MFD. We first seeded the hiPSC‐SNs in the neuronal compartment (Figure 2E, blue) for the axons to invade the cardiomyocytes compartment, before seeding hiPSC‐CMs in their compartment (Figure 2E, pink). Immunofluorescence confirmed that hiPSC‐SNs prolonged neurites through the microchannels and reached hiPSC‐CMs marked with α‐actinin (Figure 2F). The functional connection between hiPSC‐SNs and hiPSC‐CMs was previously confirmed by pharmacological application.^5^ DM1 hiPSC‐CMs exhibited variation in AP‐to‐AP time in monoculture, while co‐culture with DM1 hiPSC‐SNs increased the firing frequency of APs and decreased the beat‐to‐beat duration (Figure 2G). Interestingly, DM1 hiPSC‐CMs demonstrated pro‐arrhythmogenic phenomena, such as early or delayed afterdepolarizations (EADs/DADs) in 55% of gap‐free recordings when co‐cultured with DM1 hiPSC‐SNs, while the co‐culture control hiPSC‐SNs/control hiPSC‐CMs exhibited only 15% (Figure 2H).

This study is the first to examine the electrical activity of DM1 sympathetic neurons and their role at the NCJ using an MFD. It provided the electrophysiological basis for how neuronal and cardiomyocytes’ electrical instabilities converge to drive arrhythmogenesis in DM1 patients. APD prolongation and alternans, observed in DM1 hiPSC‐CMs, establish a vulnerable substrate characterized by marked spatial dispersion of repolarization, facilitating functional conduction blocks.^8^ Concurrently, the hyperexcitability observed in DM1 hiPSC‐SNs delivers an excessive norepinephrine release at the NCJ. This abnormal adrenergic release‐mediated calcium overload is expected to worsen cardiac calcium handling instabilities, amplifying APD alternans and triggering EADs/DADs.^9^ In addition to fibrosis observed in DM1 patients, ANS instability serves as an additional arrhythmogenic trigger for arrhythmia susceptibility in DM1 patients.^10^

However, this study has limitations, including lack of an isogenic control and the data derived from one patient with 700 CTG repeats, which does not fully capture the variability seen in DM1 patients. As such, additional studies using DM1 patients with varying CTG repeat lengths and isogenic controls are warranted.

In summary, our hiPSC‐based microphysiological system provides the first evidence of an additional direct sympathetic neuron contribution to DM1‐associated arrhythmogenesis. This system serves as a powerful platform to evaluate therapeutic strategies targeting autonomic modulations, such as stellate ganglion block, pharmacological inhibition, and gene therapy, designed to suppress sympathetic hyperactivity and restore physiological neuro‐cardiac coupling.

## AUTHOR CONTRIBUTIONS

J.B. Reisqs contributed to the conception and design of the study, analyzed and interpreted data, and wrote the first draft of the paper. Y. Sleiman contributed to analyzing and interpreting data. M. Boutjdir contributed to the design and discussion and reviewed/edited the manuscript.

## CONFLICTS OF INTEREST STATEMENT

The authors declare no conflict of interest.

## Supporting information



Supporting Information

## Data Availability

The data sets used and analyzed during the current study are available from the corresponding author upon reasonable request.
