# Supplementary material for: Sympathetic neurons’ contribution to arrhythmias in a hiPSC‐based myotonic dystrophy type 1 microphysiological system
Source: Clin Transl Med. 2026 Oct 27;16(11):e70860. doi: 10.1002/ctm2.70860 (PMC13714089; doi:10.1002/ctm2.70860)
Supplement: Supplementary file 1 [file CTM2-16-e70860-s001.docx]

**Supplemental figure 1**

**Supplemental Figure 1: Morphological maturation comparison between control and DM1 hiPSC-sympathetic neurons**

**(A)** Axonal diameter was measured in peripherin-stained control and DM1 hiPSC-SNs in control and DM1. **(B)** Axonal diameter measurement by ImageJ showed no differences between control (n=19) and DM1 (n=20) hiPSC-SNs. N= 2 independent differentiations; each point represents the mean of multiple measure from one neuron. (C-E) Fold change in gene expression analysis by RT-qPCR for beta-III tubulin (TUBB3), Peripherin (PRPH), and tyrosine hydroxylase (TH) in control and DM1 hiPSC-SNs. N= 3 independent differentiations for the control and 2 independent differentiations for the DM1 group. Mann-Whitney test.
